# MiR-34a and miR-34b/c have distinct effects on the suppression of lung adenocarcinomas

**DOI:** 10.1038/s12276-018-0203-1

**Published:** 2019-01-17

**Authors:** Jeong Seon Kim, Eun Ju Kim, Sieun Lee, Xiaochao Tan, Xin Liu, Sanghui Park, Keunsoo Kang, Jung-Sook Yoon, Yoon Ho Ko, Jonathan M. Kurie, Young-Ho Ahn

**Affiliations:** 1 Department of Molecular Medicine, College of Medicine, Ewha Womans University, Seoul, 07985 Korea; 2 Tissue Injury Defense Research Center, College of Medicine, Ewha Womans University, Seoul, 07985 Korea; 3 Department of Thoracic/Head and Neck Medical Oncology, The University of Texas MD Anderson Cancer Center, Houston, TX 77030 USA; 4 Department of Pathology, College of Medicine, Ewha Womans University, Seoul, 07985 Korea; 5 Department of Microbiology, College of Natural Sciences, Dankook University, Cheonan, Chungnam 31116 Korea; 6 Division of Oncology, Department of Internal Medicine, College of Medicine, The Catholic University of Korea, Seoul, 06591 Korea

**Keywords:** Non-small-cell lung cancer, miRNAs

## Abstract

Three miR-34 family members (miR-34a, miR-34b, and miR-34c) are clustered on two different chromosomal loci, *Mir34a* and *Mir34b/c*. These miRNAs have identical seed sequences, which are predicted to target the same set of genes. However, miR-34a and miR-34c have different sets of negatively correlated genes in lung adenocarcinoma data from The Cancer Genome Atlas. Therefore, we hypothesized that the individual miR-34 family members, which are tumor suppressive miRNAs, would have varying effects on lung tumorigenesis. To show this, we overexpressed each miR-34 cluster in murine lung cancer cells. MiR-34b/c enhanced cancer cell attachment and suppressed cell growth and invasion compared with miR-34a. In a syngeneic mouse model, both miR-34a and miR-34b/c blocked lung metastasis. However, miR-34b/c suppressed tumor growth more than miR-34a. MiR-34b/c also decreased the expression of mesenchymal markers (*Cdh2* and *Fn1*) and increased the expression of epithelial markers (*Cldn3*, *Dsp*, and *miR-200*) to a greater degree than miR-34a. These results imply that miR-34b and miR-34c inhibit epithelial-to-mesenchymal transition. Furthermore, knockout of all three miR-34 members promoted mutant *Kras*-driven lung tumor progression in mice. Similarly, lung adenocarcinoma patients with higher miR-34a/b/c levels had better survival rates than did those with lower levels. In summary, we suggest that miR-34b and miR-34c are more effective tumor suppressors than miR-34a.

## Cancer: Regulatory micro-RNAs could combine for treatment

Exploring the effects of three similar small RNA molecules called micro-RNAs (miRNAs) that can restrict the activity of specific genes reveals how they might be used in cancer treatment. RNA is best known as messenger RNA, which carries a copy of a gene’s information into the cell cytoplasm to direct protein manufacture. Many small RNAs play less well-known but crucial roles by binding to messenger RNA molecules to regulate their activity. Researchers in South Korea and USA, led by Young-Ho Ahn at Ewha Womans University in Seoul, investigated how these miRNAs can suppress lung cancer in mice. Their results reveal details of how the miRNAs inhibit the expression of specific tumor-supporting genes. They suggest that three of the RNAs administered together might treat cancer more effectively than using only one as in previous trials.

## Introduction

MicroRNAs (miRNAs) are short (20-24 nucleotides) endogenous noncoding RNAs that induce mRNA cleavage or inhibit protein translation through Watson-Crick base pairing with the 3′-untranslated regions (UTRs) of target genes^1,2^. miRNAs can suppress the expression of genes that are involved in a variety of physiologic and pathologic conditions, such as cancer development and progression^1,2^. For example, the miR-34 family members, including miR-34a, miR-34b, and miR-34c, inhibit cancer cell growth and induce apoptosis. These miRNAs function by targeting cell cycle-related genes (e.g., *CDK4, CDK6, CCND1*) and anti-apoptotic genes (e.g., *BCL2*, *SIRT1*). Therefore, they function as tumor suppressive miRNAs^3^.

MiR-34 family members are located on two separate chromosomal loci (*Mir34a* and *Mir34b/c*)^3^, implying that they could be regulated by somewhat different transcriptional and epigenetic mechanisms. For instance, miR-34b and miR-34c are upregulated during murine postnatal testicular development and spermatogenesis, while miR-34a constantly remains at a low level^4^. In non-small cell lung cancer (NSCLC), the promoter region of miR-34b/c is highly methylated compared with that of miR-34a. This methylation is a poor prognostic marker of overall survival in patients with NSCLC^5^. In addition, miR-34a and miR-34b/c do not show any correlated expression patterns in human lung cancer cells^6^. These findings suggest that although they share the same seed sequences and a common set of predicted target genes, their functional activities may differ depending on the molecular and cellular context.

In fact, several studies have shown that miRNA family members with the same seed sequence have distinct biological roles. For example, FOXP3 binds to the promoter of miR-146a but not that of miR-146b; therefore, only miR-146a mediates FOXP3-induced apoptosis in breast cancer cells^7^. Although the seed sequences of miR-200a and miR-200c differ by only a single nucleotide, these two miR-200 family members differentially regulate the morphological mode of invasion in melanoma cells^8^. In this study, we also found that miR-34a and miR-34b/c have differential effects on target gene regulation and cancer cell invasion.

To investigate the different functional roles of miR-34a and miR-34b/c in lung cancer, we analyzed their expression levels in lung cancer cell lines and in lung adenocarcinoma based on data from the Cancer Genome Atlas (TCGA). We also overexpressed each miR-34 cluster in murine lung adenocarcinoma cells that were derived from *Kras*^*G12D*^*/p53*^*R172HΔG*^ mutant mice^9^. As reported previously^6^, there was no correlation between miR-34a and miR-34b/c expression levels in lung cancer cells or in TCGA data. MiR-34b/c had more prominent suppressive effects on epithelial-to-mesenchymal transition (EMT) and cancer cell invasion than miR-34a. In addition, knockout of all three miR-34 members promoted mutant *Kras*-driven lung tumor progression in mice. This finding suggests that miR-34b and miR-34c are more potent tumor suppressors than miR-34a.

## Materials and methods

### Cell culture

Murine lung cancer cells (307P, 344LN, 344P, 344SQ, 393LN, 393P, 412P, 531LN1, 531LN2, 531LN3, 531P1, 531P2, and 713P) were isolated from *Kras*^*G12D*^*/p53*^*R172HΔG*^ mutant mice as described previously^9^. Each cell line was named according to the mouse number (e.g., 307 and 344) and the site of origin (“P” means primary lung tumors, “LN” means lymph nodes, and “SQ” means subcutaneous sites). The cells were cultured in RPMI 1640 (Welgene, Gyeongsan, Korea), which was supplemented with 10% fetal bovine serum (HyClone, Logan, UT) at 37 °C in the presence of 5% CO_2_. For the cell growth assay, the cells were plated in 24-well plates (1 × 10^4^/well) and counted after the indicated number of days using a LUNA™ automated cell counter (Logos Biosystems, Anyang, Korea). For the soft agar assay, 5 × 10^4^ cells were suspended in 0.4% agarose and seeded on 6-well plates layered with 0.8% agarose. Cell colonies were stained with nitro blue tetraziolium (0.5 mg/ml) after three weeks. For the cell attachment assay, cells were seeded on 24-well plates (5 × 10^4^/well) and incubated for 1–3 h. After washing with PBS twice, the attached cells were stained with 0.1% crystal violet; then, the optical density was measured at 595 nm. For the spheroid invasion assay, the spheroids were generated by culturing cells as hanging drops over a plate in a CO_2_ incubator for 48 h. The spheroids were embedded in collagen gels and imaged microscopically after 48 h. The invasion ratio was calculated by dividing the total invading area by the central spheroid area, which was measured using ImageJ (https://imagej.nih.gov/ij). For miR-34 overexpression, we isolated mouse miR-34a (560 bp) and miR-34b/c (1,365 bp) containing premature miRNA regions with PCR from TC-1 murine ES cell genomic DNA and ligated them into pLVX-Hyg and pLVX-Puro (Clontech, Mountain View, CA), respectively. These vectors were introduced into the cells using lentiviral infection.

### Quantitative reverse transcription-PCR (qRT-PCR)

Total RNA was isolated from cells using WelPrep™ Total RNA Isolation Reagent (Welgene). After reverse transcription with ELPis RT master mix (ELPis-Biotech, Daejeon, Korea), quantitative PCR assays were performed to analyze the mRNA levels using the BioFACT™ A-star Real-time PCR Kit including SFCgreen^®^ I (BioFACT, Daejeon, Korea). *Rpl32* was used as a reference gene, and data were analyzed using the ΔΔCt method. The qRT-PCR primers used in this study are shown in Supplementary Table 1. The microRNA levels were quantified using the HB miR Multi Assay Kit™ (HeimBiotek, Seongnam, Korea) and normalized to levels of *RNU6B* snoRNA.

### Mouse experiments

All the proposed mouse studies were approved by the Institutional Animal Care and Use Committee (IACUC) of Ewha Womans University College of Medicine and The University of Texas MD Anderson Cancer Center. The mice were cared for and euthanized according to the standards set forth by the IACUC. Syngeneic (129/Sv) mice received subcutaneous injections in the right flank of 344SQ cells (1 × 10^6^ per mouse) that were stably transduced with miR-34a, miR-34b/c, or empty lentiviral vectors. The mice were monitored daily for tumor growth. The animals were euthanized six weeks after the injection or at the first sign of morbidity. They were necropsied to isolate the primary tumors and determine the sites of metastasis. To generate miR-34-triple knockout (TKO) and *Kras*-mutant mice (129/Sv), we bred mice harboring LoxP sites surrounding *Mir34a* on chromosome 4 and the *Mir34b/c* cluster on chromosome 9 (obtained from Dr. Gerard Karsenty at Columbia University)^10^ with *Kras*-LSL mice. *Kras*-LSL mice have a *Kras*^*G12D*^ allele that is expressed under the control of a LoxP-Stop-LoxP cassette^11^. These floxed alleles were recombined in the lung epithelium by aerosolized delivery of lentiviruses expressing Cre recombinase at eight weeks of age, as previously described^11^. All mice were sacrificed by the time (36 weeks) lung cancer development was expected and necropsied to isolate the lungs for hematoxylin and eosin staining. Lung histopathology was evaluated by a board-certified pathologist (S.P.) blinded to the mouse genotypes.

### RNA sequencing

The RNA quality was assessed by analysis of rRNA band integrity on an Agilent RNA 6000 Nano system (Agilent Technologies, Santa Clara, CA). Before cDNA library construction, 2 μg of total RNA and magnetic beads with oligo-dT were used to enrich poly-A mRNA. Then, the purified mRNAs were disrupted into short fragments, and the double-stranded cDNAs were immediately synthesized. The cDNAs were subjected to end-repair and poly-A addition and connected with sequencing adapters using the TruSeq RNA sample prep kit (Illumina, San Diego, CA). The suitable fragments automatically purified by the BluePippin 2% agarose gel cassette (Sage Science, Beverly, MA) were selected as templates for PCR amplification. The final library sizes and qualities were evaluated electrophoretically with an Agilent High Sensitivity DNA kit (Agilent Technologies), and the fragment was found to be between 350–450 bp. Subsequently, the library was sequenced using an Illumina HiSeq2500 sequencer (Illumina). Then, RNA sequencing data were analyzed using Octopus-toolkit^12^.

### Statistical analysis

Data were analyzed with Student’s *t*-tests, Spearman’s rank correlation tests, Fisher’s exact tests, and log-rank tests using GraphPad Prism (San Diego, CA).

## Results

### miR-34a and miR-34b/c have distinct expression patterns in lung adenocarcinomas

**Fig. 1:**
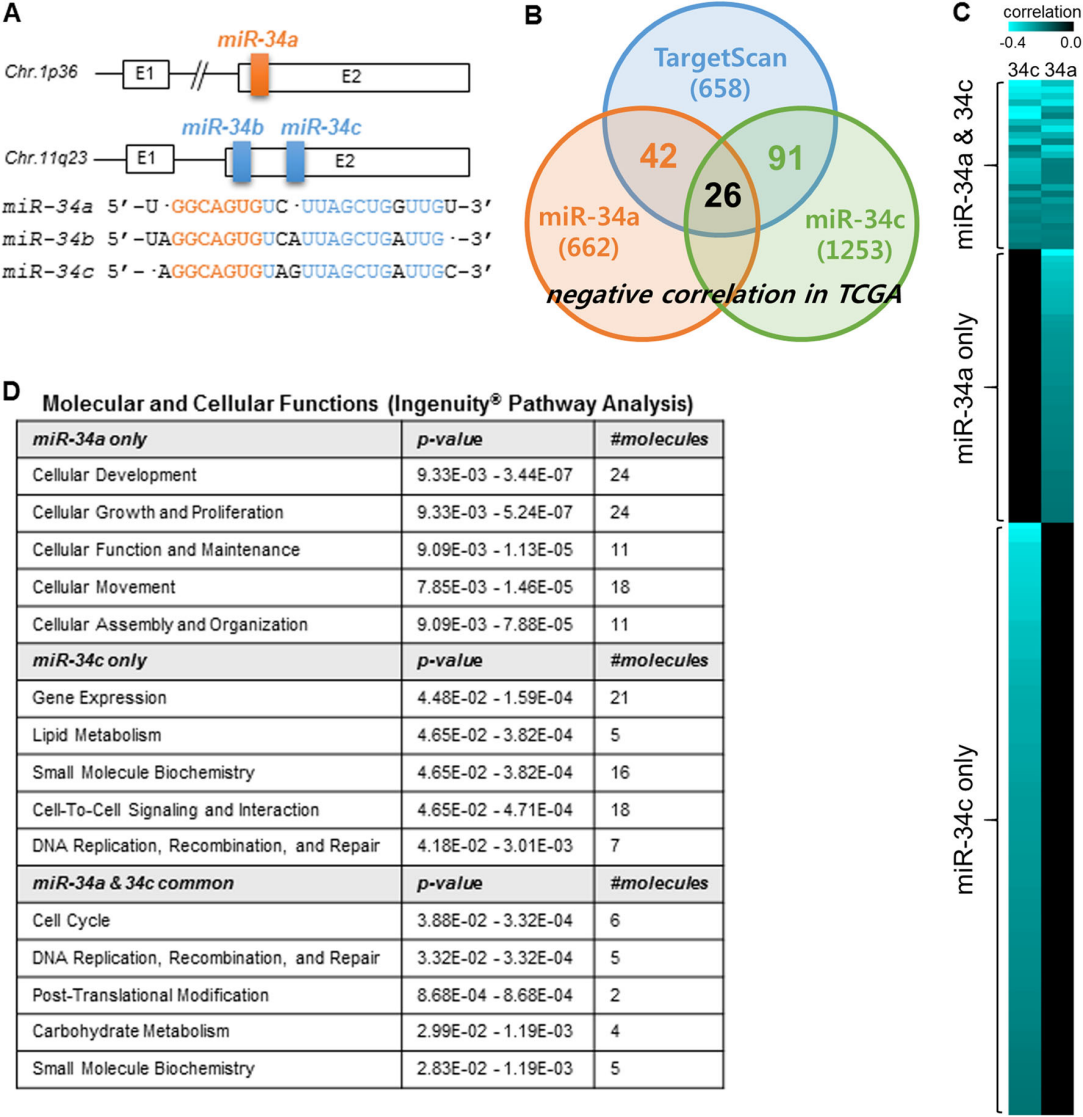
MiR-34a and miR-34b/c have distinct predicted target genes. **a** Chromosomal loci of miR-34 family members. Primary miR-34a (pri-miR-34a; white rectangular boxes) is located on chromosome 1, while pri-miR-34b/c is on chromosome 11. The colored boxes (orange or blue) indicate premature miRNAs. Mature miR-34 sequences are shown in the diagram. Seed sequences are indicated in orange. **b** Prediction of miR-34 target genes. Target genes of miR-34a and miR-34c were predicted using TargetScan (http://www.targetscan.org). We isolated the negatively correlated genes either with miR-34a or with miR-34c in lung adenocarcinoma data from TCGA (miRGator v3.0, http://mirgator.kobic.re.kr). The combined results are presented in the Venn diagram. **c** Heatmap of target genes predicted in (**b**). Genes negatively correlated with miR-34a only, with miR-34c only, or with both miR-34a and miR-34c are presented. The blue color intensity is proportional to the correlation coefficients. **d** Ingenuity^®^ Pathway Analysis (molecular and cellular functions) of the miR-34 target genes

Three miR-34 family members are clustered on two different chromosomal loci, *Mir34a* and *Mir34b/c*. These miRNAs have identical seed sequences and therefore may share a common set of target genes (Fig. 1a). We used TargetScan (http://www.targetscan.org) and lung adenocarcinoma data from TCGA to predict the target genes of miR-34 (Fig. 1b). In TargetScan, a total of 658 genes were predicted as putative targets of both miR-34a and miR-34c. In the data from TCGA, the expression levels of 662 and 1253 genes were negatively correlated with the expression levels of miR-34a and miR-34c, respectively (miRGator v3.0, http://mirgator.kobic.re.kr)^13^. After combining these lists, we predicted 68 target genes of miR-34a and 117 target genes of miR-34c (Fig. 1b, c). Interestingly, most of these genes did not overlap. The Ingenuity^®^ Pathway Analysis of each gene set also produced different results (Fig. 1d). These findings suggest that miR-34a and miR-34b/c function differently in an in vivo cellular context.

**Fig. 2:**
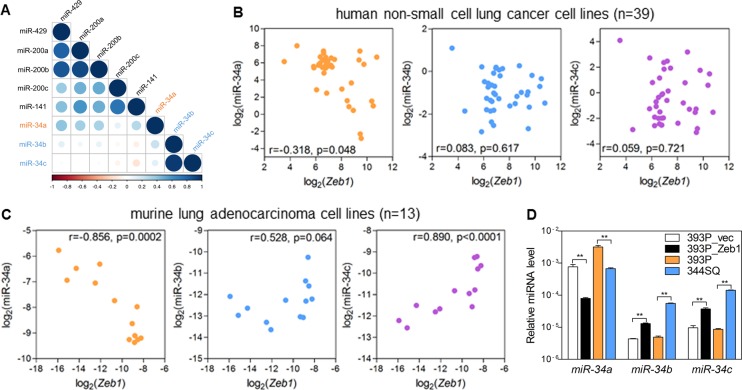
miR-34a and miR-34b/c have different expression patterns in lung adenocarcinomas. **a** Correlation matrix of miR-34 and miR-200 expression levels obtained from lung adenocarcinoma data from TCGA data. Positive correlations are in blue, and negative correlations are in red. Color intensity and circle size are proportional to the correlation coefficients. **b** Scatter plots of *Zeb1* mRNA and miR-34 family members using quantitative reverse transcription PCR (qRT-PCR) data from 39 human non-small cell lung cancer cell lines. Spearman’s correlation *r*- and *p-*values are denoted. **c** Scatter plots of *Zeb1* mRNA and miR-34 family members using qRT-PCR data from 13 murine lung adenocarcinoma cell lines. Spearman’s correlation *r*- and *p-*values are denoted. **d** qRT-PCR of miR-34 family members (miR-34a, miR-34b, and miR-34c) in Zeb1- or empty vector (vec)-transduced 393P, parental 393P, and 344SQ cells. Expression levels were normalized to the *RNU6B* snoRNA level. Data are expressed as the mean ± SD (*n* = 3). **P ≤ 0.01; two-tailed Student’s *t* test

In the lung adenocarcinoma data from TCGA, unlike the miR-200 family members, the miR-34 family members did not have positively correlated expression levels (Fig. 2a). Only the miR-34a levels correlated positively with miR-200 levels. This finding implies that the two miR-34 clusters are under different transcriptional regulation. Previously, we reported that miR-34a is negatively regulated by Zeb1, an EMT-inducing transcription factor. In human non-small cell lung cancer cell lines (Fig. 2b) and murine lung adenocarcinoma cell lines derived from *Kras*/*p53*-double mutant mice (Fig. 2c)^6^, miR-34a was negatively correlated with *Zeb1* mRNA levels. However, miR-34b and miR-34c were not. Clearly, miR-34b and miR-34c have a strong positive correlation with each other because they are in the same cluster (Fig. 2a; Fig. S1A-C). In epithelial-like 393P murine lung cancer cells, the ectopic expression of Zeb1 suppressed miR-34a, while it slightly increased miR-34b/c expression. In addition, mesenchymal-like 344SQ cells expressed less miR-34a and more miR-34b/c than 393P cells (Fig. 2d). All these data suggest that miR-34a and miR-34b/c are regulated by different transcriptional mechanisms and have different biological functions.

### MiR-34a and miR-34b/c have different effects on the characteristics of cancer cells

**Fig. 3:**
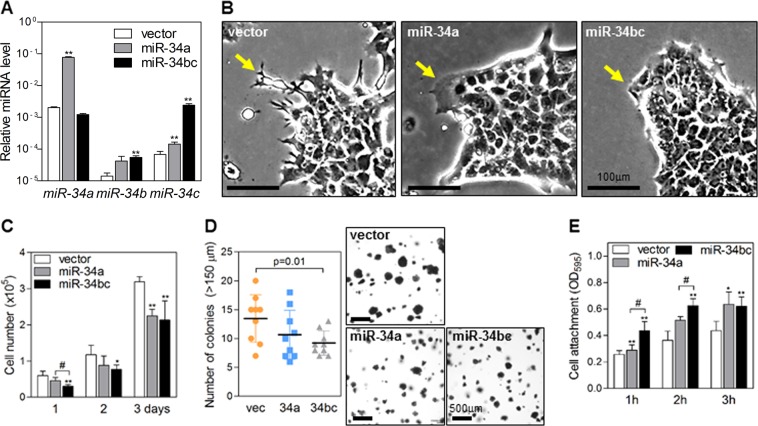
miR-34b/c enhances the anchorage- and attachment-dependence of lung cancer cells. **a** qRT-PCR of miR-34 family members in 344SQ cells transduced with miR-34a, miR-34b/c, or empty vectors. The expression levels were normalized to the *RNU6B* snoRNA level. Data are expressed as the mean ± SD (*n* = 3). **P ≤ 0.01; two-tailed Student’s *t* test. **b** Phase-contrast microscopy images of 344SQ cells transduced with miR-34a, miR-34b/c, or empty vectors. Arrows indicate filopodia formation changes at the leading edge. **c** Growth of miR-34-transduced 344SQ cells. Cells were seeded on 24-well plates. Cell numbers were counted after the indicated days. Data are expressed as the mean ± SD (*n* = 4). *P ≤ 0.05, **P ≤ 0.01 compared with vector and #*P* < 0.05 compared with miR-34a cells; two-tailed Student’s *t* test. **d** Soft agar colony formation assay in 344SQ cells transduced with miR-34a (34a), miR-34b/c (34bc), or an empty vector (vec). Cells seeded in soft agar were stained with nitro blue tetrazolium 3 weeks after seeding. Colonies larger than 150 μm in diameter were counted (left graph). Data are expressed as the mean ± SD (*n* = 9). *P*-values were determined by two-tailed Student’s *t* test. **e** Cell attachment assay in miR-34-transduced 344SQ cells. Attached cells at the bottom of the culture plates were quantified 1, 2, or 3 h after seeding by optical densitometry (595 nm) of the cells stained with crystal violet. Data are expressed as the mean ± SD (*n* = 4). *P ≤ 0.05, **P ≤ 0.01 compared with the vector and #*P* ≤ 0.05 compared with miR-34a cells; two-tailed Student’s *t* test

We suspected that miR-34a and miR-34b/c have different biological functions. To show this, we overexpressed each cluster in 344SQ cells (Fig. 3a). As reported earlier^6^, both miR-34a and miR-34b/c suppressed filopodia formation at the leading edge of migrating cells (arrows in Fig. 3b). Both miR-34a and miR-34b/c also inhibited cancer cell growth; however, the growth inhibitory effect of miR-34b/c was greater than that of miR-34a (Fig. 3c). In soft agar cultures, only miR-34b/c suppressed anchorage-independent growth (Fig. 3d). Furthermore, miR-34b/c-transduced cells attached to the bottom of culture dishes faster than miR-34a-transduced cells (Fig. 3e). These findings suggest that miR-34b/c enhances the anchorage- and attachment-dependence of lung cancer cells.

**Fig. 4:**
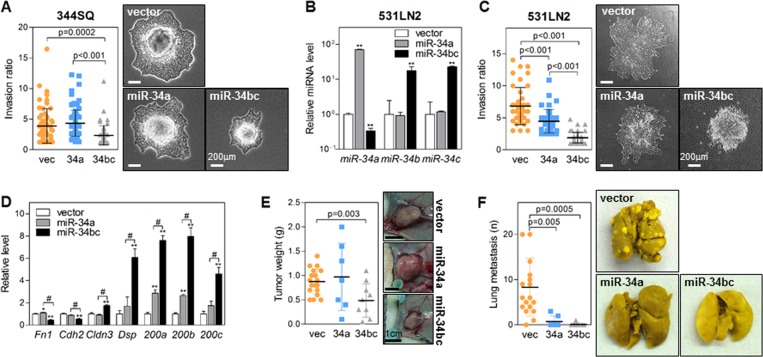
MiR-34b/c inhibits invasion and EMT more effectively than miR-34a. **a** Spheroid invasion assay in miR-34-transduced 344SQ cells. Spheroids were seeded on collagen gels and then cultured for 48 h. Data are expressed as the mean ± SD (vec, *n* = 67; 34a, *n* = 96; 34bc, *n* = 59). *P*-values were determined using the two-tailed Student’s *t* test. **b** qRT-PCR of miR-34 family members in miR-34-transduced 531LN2 cells. Expression levels were normalized to the *RNU6B* snoRNA level, and the values relative to those of the 531LN2-vector (set at 1.0) are presented. Data are expressed as the mean ± SD (*n* = 3). **P ≤ 0.01; two-tailed Student’s *t* test. **c** Spheroid invasion assay in miR-34-transduced 531LN2 cells. Spheroids were seeded on collagen gels and then cultured for 48 h. Data are expressed as the mean ± SD (vec, *n* = 41; 34a, *n* = 41; 34bc, *n* = 58). *P*-values were determined using two-tailed Student’s *t* test. **d** qRT-PCR of EMT markers (*Fn1* and *Cdh2*: epithelial, *Cldn3* and *Dsp*: mesenchymal markers) and miR-200 family members (miR-200a, miR-200b, and miR-200c) in miR-34-transduced 344SQ cells. Expression levels were normalized to *Rpl32* (for mRNA) or *RNU6B* snoRNA (for miRNA) levels. The values relative to those of the 344SQ vector (set at 1.0) are presented. Data are expressed as the mean ± SD (*n* = 3). *P ≤ 0.05, **P ≤ 0.01 compared with vector and #*P* ≤ 0.01 compared with miR-34a cells; two-tailed Student’s *t* test. **e**, **f** Primary tumor weight (**e**) and number of lung metastases (**f**) in syngeneic mice (129/Sv) injected subcutaneously with miR-34-transduced 344SQ cells (1 × 10^6^ cells per mouse). Lungs were stained with Bouin’s solution to visualize the metastatic tumor nodules. Data are expressed as the mean ± SD (vec, *n* = 19; 34a, *n* = 7; 34bc, *n* = 10). *P*-values were determined using two-tailed Student’s *t* test

Next, to investigate the effect of miR-34 on cancer cell invasion, we performed collagen gel invasion assays using cell spheroids cultured in hanging drops^14^. Unlike miR-34a, miR-34b/c significantly suppressed the expansion and invasion of cancer cell spheroids in collagen gels (Fig. 4a). Similar results were observed in another murine lung cancer cell line, 531LN2 (Fig. 4b, c). MiR-34a slightly inhibited cancer cell invasion compared with an empty vector. However, miR-34b/c had a more potent suppressive effect than miR-34a (Fig. 4c). Epithelial cancer cells generally acquire invasiveness via EMT^15^; therefore, we determined the EMT status of miR-34-transduced cells by measuring the mRNA expression of EMT markers (Fig. 4d). MiR-34b/c decreased the expression of mesenchymal markers (*Fn1* and *Cdh2*) and increased the expression of epithelial markers (*Cldn3* and *Dsp*). In contrast, miR-34a had little or no effect on these markers (Fig. 4d). The miR-200 family members (miR-200a, 200b, and 200c), which are EMT-inhibitory miRNAs, were more strongly induced by miR-34b/c than they were by miR-34a (Fig. 4d). This finding suggests that miR-34b/c is a stronger EMT suppressor than miR-34a.

In addition, we injected miR-34a- or miR-34b/c-overexpressing cells into syngeneic mice to compare the effects of these miRNAs on tumor growth and metastasis. MiR-34b/c inhibited primary tumor growth at the injection sites more than miR-34a (Fig. 4e). However, there was no significant difference in lung metastasis inhibition (Fig. 4f). Based on these findings, we conclude that miR-34a and miR-34b/c have different effects on the characteristics of cancer cells. In particular, miR-34b and miR-34c are more potent tumor suppressive miRNAs than miR-34a.

### MiR-34a and miR-34b/c have different effects on the regulation of target gene expression

**Fig. 5:**
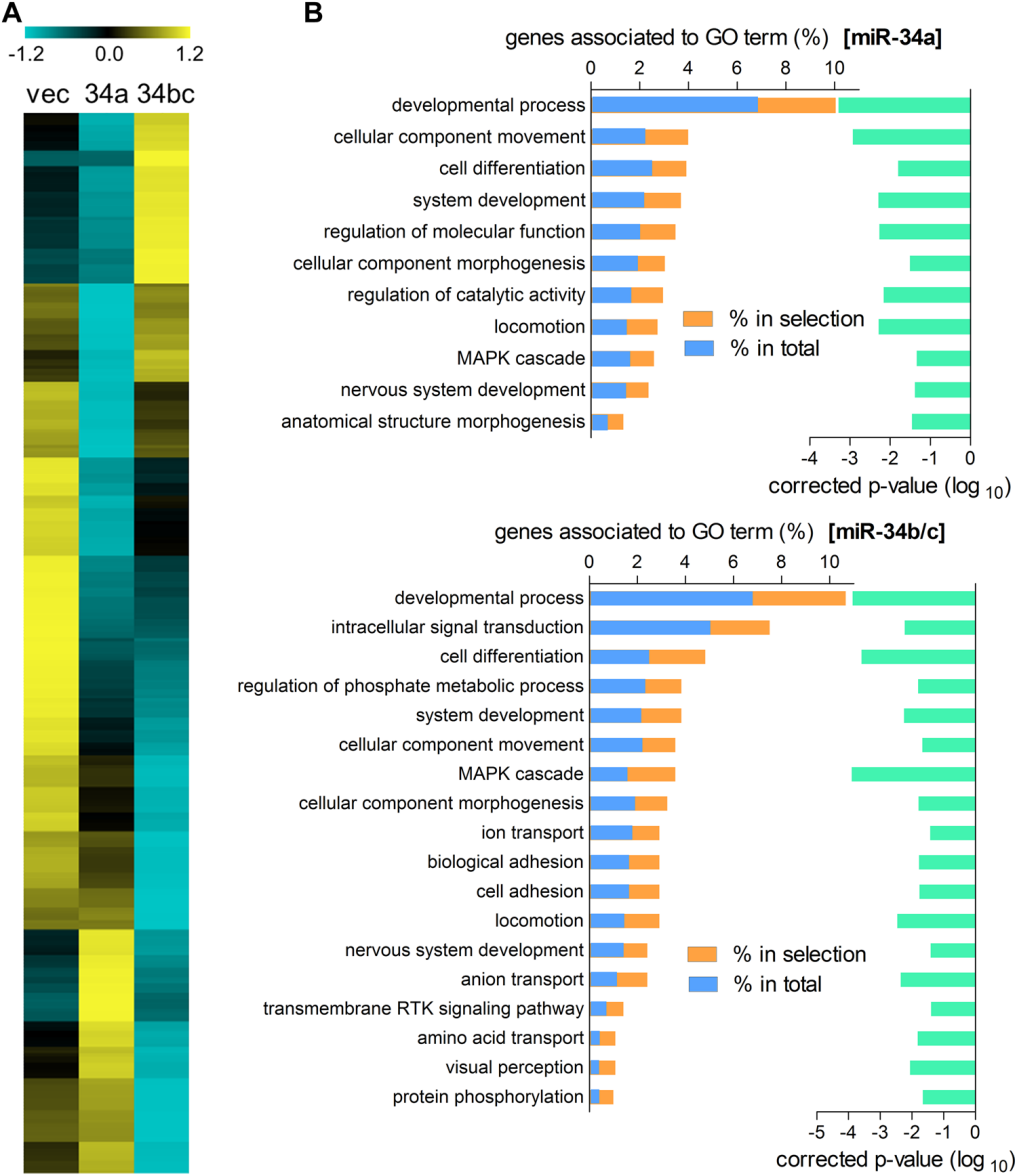
MiR-34a and miR-34b/c have different effects on target gene expression. **a** Heatmap of differentially expressed genes (fold change ≥ 2) in miR-34-transduced 344SQ cells compared with those of vector-transduced cells, as identified by RNA sequencing. Yellow: increased expression; blue: decreased expression. **b** Gene ontology (GO) enrichment analysis (http://pantherdb.org) of differentially expressed genes in the miR-34- or miR-34b/c-transduced 344SQ cells. Genes associated with the GO terms are shown in the left hand bars for differentially expressed genes (% in selection, orange) and for the total set of 22,262 genes (% in total, blue). The adjusted *p*-values (false discovery rate, FDR) are represented in the right hand bars (green). The GO terms with fold-enrichment ≥ 1.5 and FDR ≤ 0.05 were selected for the results

Next, we sought to determine whether miR-34a and miR-34b/c have distinct effects on target gene expression. To do so, we used RNA sequencing to profile the entire mRNA transcriptomes of 344SQ cells that were transduced with miR-34a, miR-34b/c, or empty vectors. As expected, the mRNA expression patterns were significantly different between miR-34a- and miR-34b/c-overexpressing cells (Fig. 5a). Although many genes were modulated in a similar fashion by miR-34a and miR-34b/c, the expression of numerous genes was specifically changed by only miR-34a or miR-34b/c. In the gene ontology (GO) enrichment analysis (http://pantherdb.org), genes associated with GO terms such as “biological adhesion (*P* = 1.74E-02)” and “cell adhesion (*P* = 1.67E-02)” were modulated exclusively by miR-34b/c (Fig. 5b). This result corresponds to the finding that miR-34b/c increases cell adhesion dependency (Fig. 3d, e).

To understand the cause of differential target gene regulation by miR-34a and miR-34b/c, we predicted the 2D structures of miRNA/mRNA complexes using VfoldCPX software (http://rna.physics.missouri.edu/vfoldCPX). The 3′-UTRs of the miR-34b/c-specific genes *Hk1* and *Mmab* (Fig. S2A) were predicted to form similar duplex structures with miR-34b and miR-34c. These structures were quite distinctive from those with miR-34a (Fig. S2B). Ultimately, more detailed studies are needed; however, our results suggest that miR-34a and miR-34b/c can regulate target genes through different mechanisms.

### MiR-34a and miR-34b/c have suppressive effects on spontaneous tumor development in mice

**Fig. 6:**
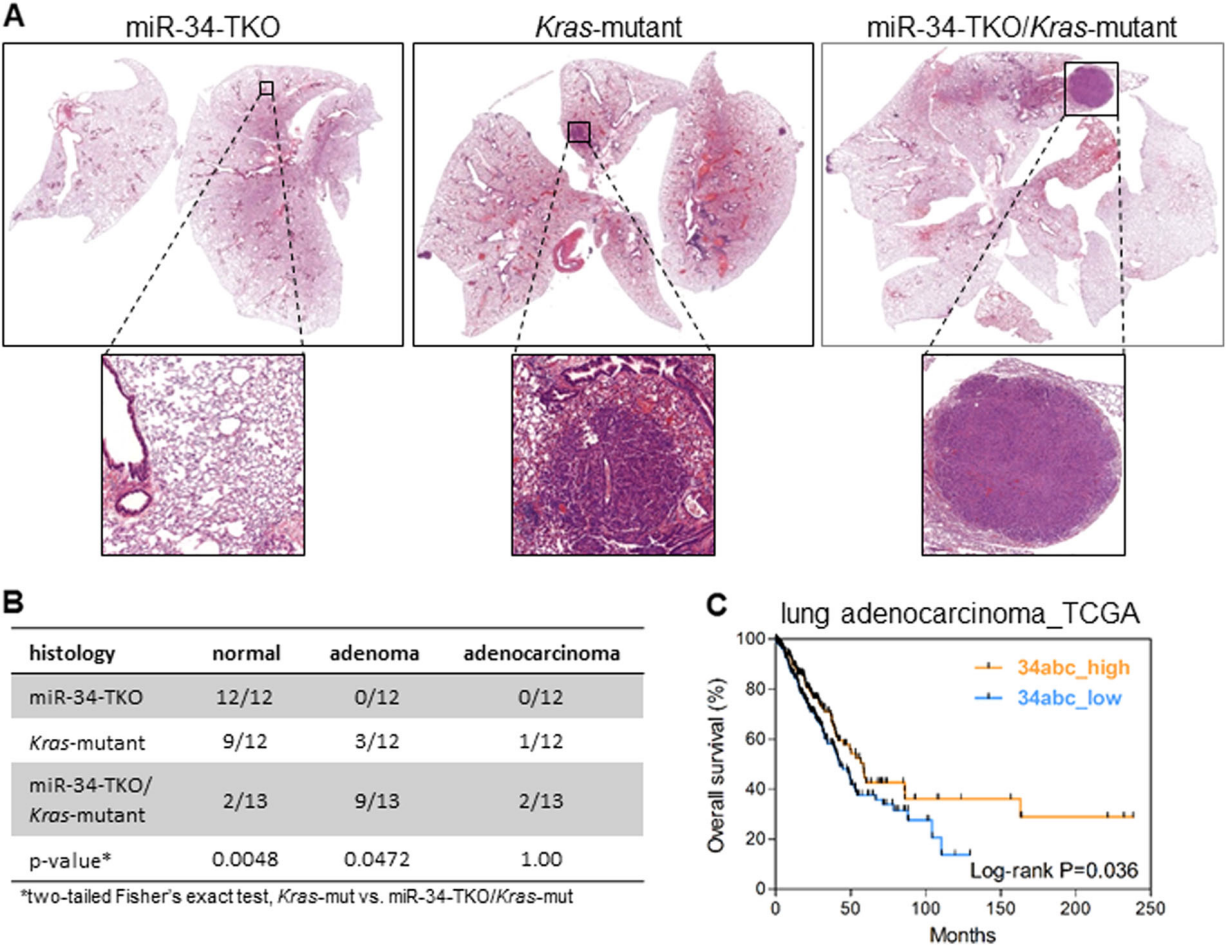
MiR-34-triple knockout enhances mutant *Kras*-driven lung tumorigenesis. **a** Histologic sections of lung tissues from the miR-34-triple knockout (TKO), *Kras*-mutant (*Kras*^*G12D*^), and miR-34-TKO/*Kras*-mutant mice. Lung tissue sections from each cohort were stained with hematoxylin and eosin and photographed. Magnifications of the adenomas are shown in the insets. **b** Lung histopathology of miR-34-TKO, *Kras*-mutant, and miR-34-TKO/*Kras*-mutant mice. *P*-values were determined using two-tailed Fisher’s exact tests. **c** Kaplan–Meier plot of lung adenocarcinoma patients from TCGA data. Patients were divided into two groups (high and low) based on the total miR-34 (miR-34a, miR-34b, and miR-34c) expression levels. *P*-values were determined with the log-rank test

Because miR-34a and miR-34b/c have different tumor suppressive effects, we hypothesized that the deletion of both genomic clusters would further promote tumorigenesis in a lung cancer mouse model. To test this, we bred *Kras*-LSL (LoxP-Stop-LoxP) mice harboring the *Kras* mutation (*Kras*^*G12D*^)^11^ with miR-34-triple knockout (TKO) mice^16^. In the TKO mice, all three miR-34 members were deleted using the Cre-LoxP system after intratracheal injection of Cre lentivirus (Fig. S3A and B). Histopathological analysis of the lungs demonstrated that the combination of the *Kras* mutation and miR-34-TKO increased the incidence of lung adenoma compared with the *Kras* mutation or miR-34-TKO alone (Fig. 6a, b). This finding suggests that miR-34-TKO further promotes lung cancer development driven by the *Kras* mutation. The analysis of data from lung adenocarcinoma patients from TCGA similarly revealed that the survival rate was greater in patients with higher expression levels of all three miR-34 members than it was in those with lower expression (Fig. 6c). Based on these data, we conclude that the combination of miR-34a and miR-34b/c inhibits lung cancer development.

## Discussion

Our findings indicate that miR-34a and miR-34b/c are transcriptionally regulated by different mechanisms. These miRNAs also have differential anti-tumor potentials in lung adenocarcinoma cells. We previously found that miR-34a is negatively correlated with *Zeb1* mRNA and positively correlated with miR-200 family members in lung adenocarcinoma data from TCGA and lung cancer cells^6^. However, there was no correlation between miR-34b/c and either *Zeb1* mRNA or miR-200. There was also no significant correlation observed between miR-34a and miR-34b/c. In contrast, there is a strong positive correlation between miR-34b and miR-34c because they are expressed from the same primary transcript. ΔNp63 is a transcriptional target of Zeb1, which in turn transactivates miR-34a but not miR-34b/c^6^. This may explain why only miR-34a correlates negatively with *Zeb1*. Regardless, the miR-34b/c-specific transcription mechanisms remain poorly understood. Differential promoter methylation may result in the distinct expression patterns of miR-34a and miR-34b/c^5^. However, further studies are needed to clarify the transcriptional regulation that occurs across miR-34 family members.

In most assays performed in this study, miR-34b/c had more potent anti-tumor activity than miR-34a. These assays included cell growth on culture dishes or in soft agar, adhesion to culture dishes, invasion into collagen gels, expression of EMT makers, and tumor growth in syngeneic mice. Although the same set of target genes was predicted in TargetScan, there is no notable overlap between the genes that are negatively correlated with miR-34a and miR-34c in TCGA data. In addition, in the mRNA profiling, we found that many genes were only influenced by either miR-34a or miR-34b/c, but not by both. All these findings suggest that miR-34a and miR-34b/c have specific and distinct gene targets in vivo, despite their shared seed sequence.

The miRNA seed sequences are critical for the miRNA:mRNA interaction. These sequences are used to predict the miRNA target genes in most algorithms^17–19^. Other regions of miRNAs, however, have also been shown to play important roles in the interaction with target mRNAs. In a Drosophila study, the miRNA 3′-ends were suggested to be the key determinants of target specificity across miRNA family members with the same seed sequences^20^. The “centered sites” in the middle region of miRNAs have also been shown to be functional during mRNA pairing^21^. Although miR-34a and miR-34b/c have identical seed sequences, their putative targets and cellular functions are quite different. The RNA folding structures of the miRNA:target gene duplexes were also different between miR-34a and miR-34b/c. This finding implies that other factors besides the seed sequence influence the target specificity.

miR-34a is one of the most widely studied tumor suppressive miRNAs. Interestingly, however, knockout (KO) of miR-34a alone had little or no effect on tumorigenesis in mice^22,23^. It was only when these mice were bred with other mice harboring oncogenic variations (such as the *Kras*^*G12D*^ mutation and the *SmoA1* transgene) that miR-34a-KO promoted tumorigenesis^22,23^. Furthermore, it was only when all three miR-34 members were deficient that the pluripotent ability of stem cell-generating mouse embryonic fibroblasts was enhanced^24^. In addition, in a colon cancer mouse model with the APC mutation (*Apc*^*Min/+*^), neither miR-34a- nor miR-34b/c-deletion had a significant effect; however, the combined deletion of all three miR-34 members enhanced *Apc*^*Min/+*^-induced intestinal tumorigenesis^25^. Here, we found that the KO of both miR-34a and miR-34b/c could promote lung tumorigenesis driven by the *Kras* mutation. This finding further supports the hypothesis that the combination of miR-34a and miR-34b/c has a suppressive effect on tumor development. Given that miR-34a was one of the first miRNAs included in clinical trials for solid tumors, myeloma, and lymphoma^26,27^, combinational treatment for miR-34a and miR-34b/c would be more beneficial for cancer patients than would treatment for miR-34a alone.

## Supplementary information

Supplementary Data
