# Supplementary material for: MiR-34a and miR-34b/c have distinct effects on the suppression of lung adenocarcinomas
Source: Exp Mol Med. 2019 Jan 17;51(1):9. doi: 10.1038/s12276-018-0203-1 (PMC6353903; doi:10.1038/s12276-018-0203-1)
Supplement: Supplementary file 1 — Supplementary Data [file 12276_2018_203_MOESM1_ESM.pdf]

Supplementary Table 1. qRT-PCR primers used in this study.

Gene	Forward	Reverse
Rpl32	5'-GGAGAAGGTTCAAGGGCCAG-3'	5'-TGCTCCCATAACCGATGTTGG-3'
Fn1	5'-CCCTATCTCTGATACCGTTGTCC-3'	5'-TGCCGCAACTACTGTGATTCGG-3'
Cdh2	5'-CCTCCAGAGTTTACTGCCATGAC-3'	5'-CCACCACTGATTCTGTATGCCG-3'
Cldn3	5'-CACCTGACTACCGGGCCTAG-3'	5'-GGTTTCTTTGTCCATTCGGCT-3'
Dsp	5'-GGTACGTGACGGGCCCAGGA-3'	5'-GGCCCACGGAAGGGACAAGC-3'
Hk1	5'-GAAAGGAGACCAACAGCAGAGC-3'	5'-TTCGTTCCTCCGAGATCCAAGG-3'
Mmab	5'-GTGGTGCCTCTTGTCCAGATGG-3'	5'-TCTCTTGGCTGCCCTCCTTCAT-3'

Supplementary Figure Legends

Supplementary Figure 1. miR-34a and miR-34b/c show no correlated expression patterns in lung adenocarcinomas.

A. Scatter plots between miR-34 family members using human lung adenocarcinoma TCGA data (n=489). Spearman correlation r and p values are denoted.
B. Scatter plots between miR-34 family members using qRT-PCR data from 39 human non-small cell lung cancer cell lines. Spearman correlation r and p values are denoted.
C. Scatter plots between miR-34 family members using qRT-PCR data from 13 murine lung adenocarcinoma cell lines. Spearman correlation r and p values are denoted.

Supplementary Figure 2. miR-34a and miR-34b/c are predicted to form different duplex structures with target genes.

A. qRT-PCR of miR-34b/c-specific target genes (*Hk1* and *Mmab*) in miR-34-transduced 344SQ cells. Expression levels were normalized to the *Rpl32* level, and then relative values to those of 344SQ-vector (set at 1.0) were presented. Data are expressed as mean+SD (n=3). **P≤0.01 compared with vector and #P≤0.05, ##P≤0.01 compared with miR-34a cells; two-tailed Student's t test.
B. Predicted 2D structures of miRNA/mRNA duplex using VfoldCPX software (http://rna.physics.missouri.edu/vfoldCPX). 3'-UTR's of miR-34b/c-specific genes, *Hk1* and *Mmab* (orange), and miR-34 family members (blue) are demonstrated in the hybrid duplex models.

Supplementary Figure 3. Generation of miR-34-triple knockout and *Kras*-mutant mice.

A. Diagram shows that *Kras*-LSL (LoxP-Stop-LoxP) mice harboring the *Kras* mutation (*Kras*^{G12D}) were bred with miR-34-triple knockout (TKO) mice (in whom all three miR-34 members were deleted by Cre-LoxP system after intratracheal injection of Cre lentivirus).
B. Genotyping of *Kras*-LSL, miR-34a-flox, and miR-34bc-flox mice. Wildtype (wt), heterozygote- (wt/fl), and homozygote-floxed (fl/fl) mice were identified by using specific PCR primer sets.

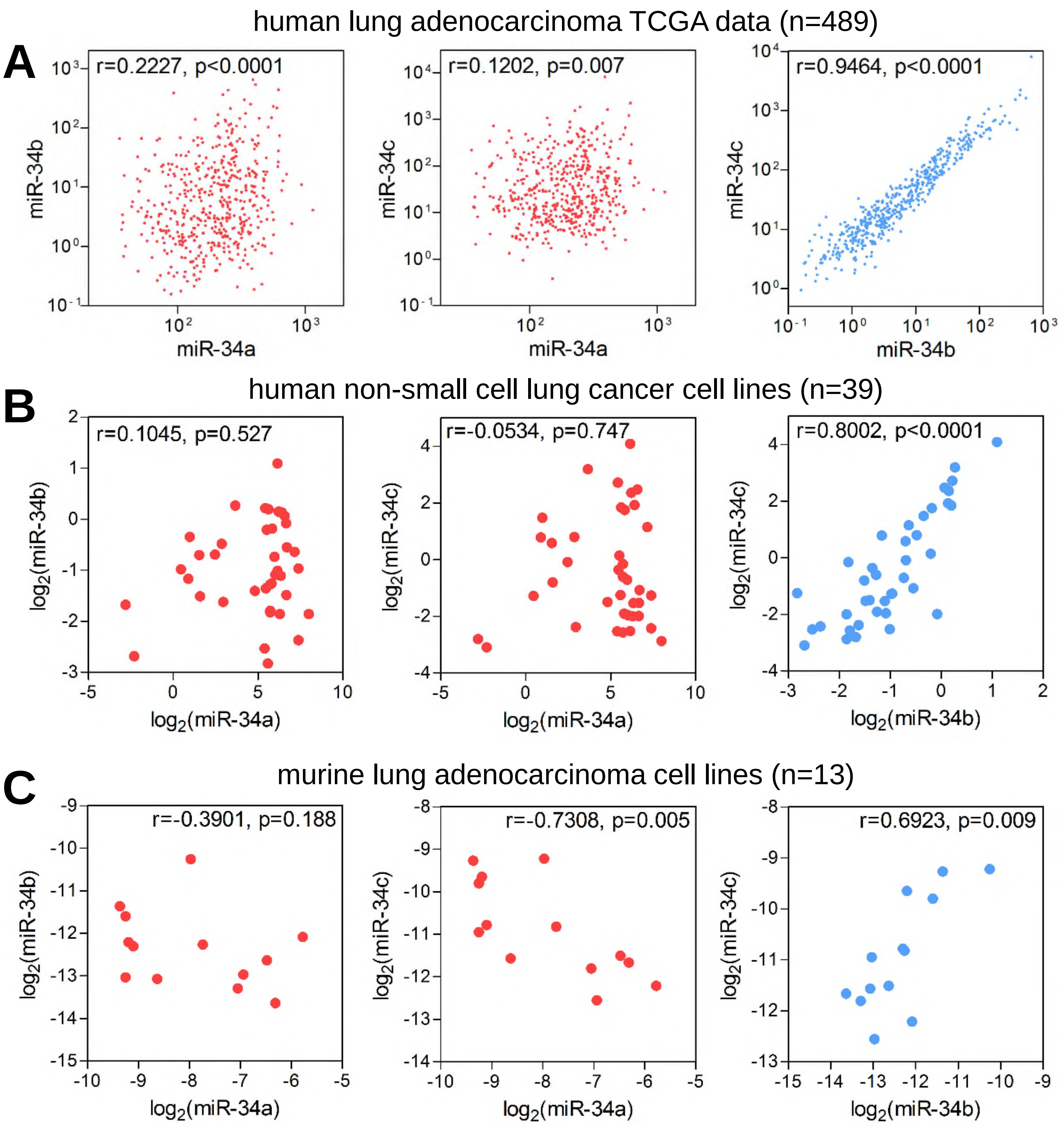

Supplementary Figure 1

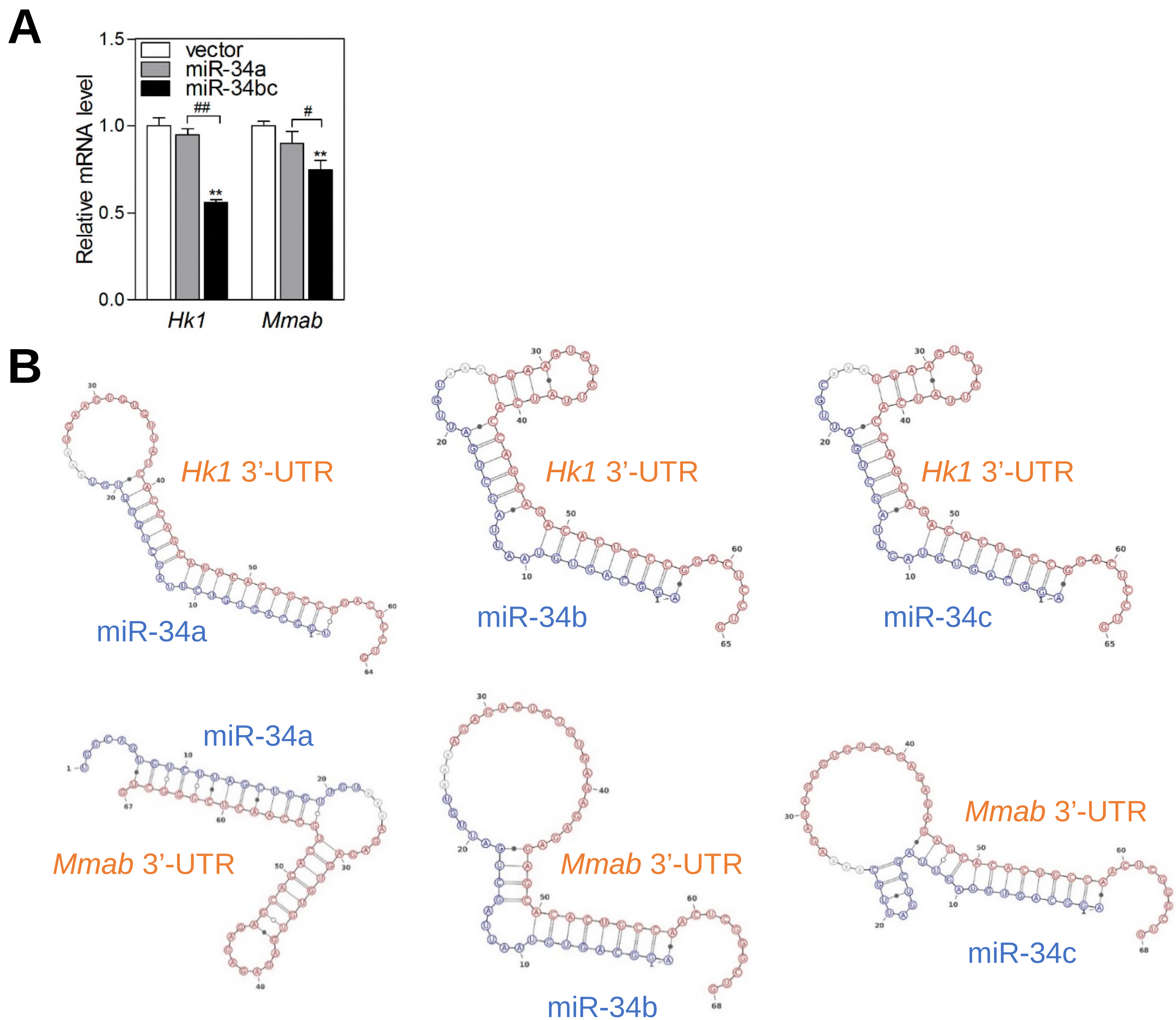

Supplementary Figure 2

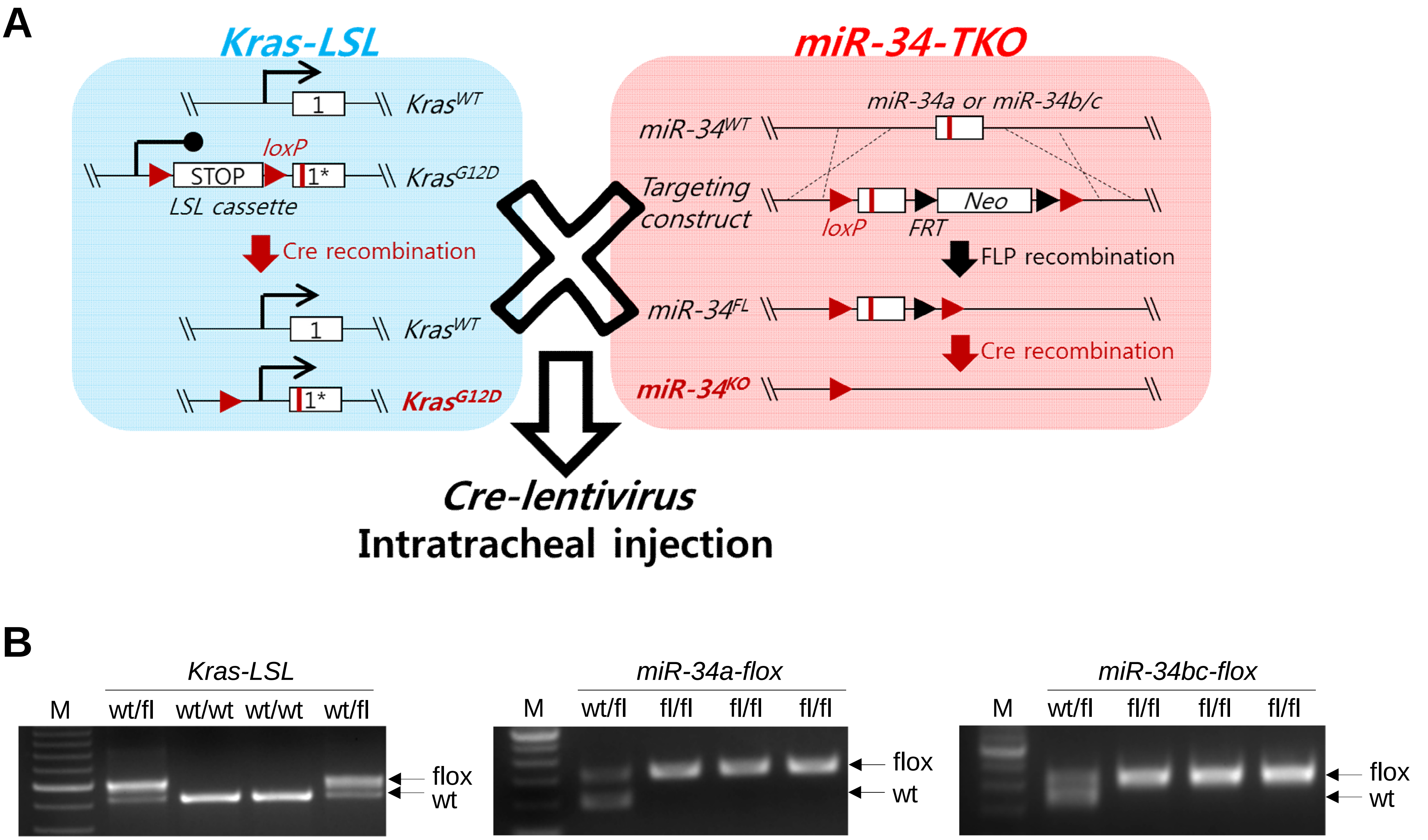

Supplementary Figure 3
